# Properties of pattern standard deviation in open-angle glaucoma patients with hemi-optic neuropathy and bi-optic neuropathy

**DOI:** 10.1371/journal.pone.0171960

**Published:** 2017-03-01

**Authors:** Dong Won Heo, Kyoung Nam Kim, Min Woo Lee, Sung Bok Lee, Chang-sik Kim

**Affiliations:** Department of Ophthalmology, Chungnam National University Hospital, Chungnam National University School of Medicine, Daejeon, Korea; Bascom Palmer Eye Institute, UNITED STATES

## Abstract

**Purpose:**

To evaluate the properties of pattern standard deviation (PSD) according to localization of the glaucomatous optic neuropathy.

**Methods:**

We enrolled 242 eyes of 242 patients with primary open-angle glaucoma, with a best-corrected visual acuity ≥ 20/25, and no media opacity. Patients were examined via dilated fundus photography, spectral-domain optical coherence tomography, and Humphrey visual field examination, and divided into those with hemi-optic neuropathy (superior or inferior) and bi-optic neuropathy (both superior and inferior). We assessed the relationship between mean deviation (MD) and PSD. Using broken stick regression analysis, the tipping point was identified, i.e., the point at which MD became significantly associated with a paradoxical reversal of PSD.

**Results:**

In 91 patients with hemi-optic neuropathy, PSD showed a strong correlation with MD (r = −0.973, β = −0.965, p < 0.001). The difference between MD and PSD (“−MD−PSD”) was constant (mean, −0.32 dB; 95% confidence interval, −2.48~1.84 dB) regardless of visual field defect severity. However, in 151 patients with bi-optic neuropathy, a negative correlation was evident between “−MD−PSD” and MD (r^2^ = 0.907, p < 0.001). Overall, the MD tipping point was −14.0 dB, which was close to approximately 50% damage of the entire visual field (p < 0.001).

**Conclusions:**

Although a false decrease of PSD usually begins at approximately 50% visual field damage, in patients with hemi-optic neuropathy, the PSD shows no paradoxical decrease and shows a linear correlation with MD.

## Introduction

In glaucoma patients, visual field deterioration is one of the most important indicators in initiating or strengthening a glaucoma treatment strategy [1, 2]. Global indices, including mean deviation (MD) and pattern standard deviation (PSD) provided by a Humphrey visual field analyzer (Carl Zeiss Meditec, Dublin, CA, USA), a standard static perimeter, can help to determine the possible visual field deterioration. MD is the weighted mean value of all test points in the total deviation plot, which is based on the deviation from age-matched normal values. Although MD is a useful indicator of total depression in visual field sensitivity that shows linear change according to glaucoma progression [3, 4], generalized depression can result not only from glaucoma but also from media opacity, such as a cataract, or decreased retinal sensitivity, such as high myopia [5–12].

PSD values are calculated based on the variation from the normal, age-corrected hill of vision involving the total deviation plot. PSD is a metric that indicates a difference in the sensitivity of adjacent tested points. In glaucoma patients, as irregular depression of visual field sensitivity progresses, PSD values increase. However, as visual field damage progresses to the point of causing an overall reduction in sensitivity, PSD values decrease. Hence, PSD is considered an inappropriate parameter for determining the stage of glaucoma [13].

Regarding localization of glaucomatous optic neuropathy, some patients have bioptic neuropathy, superior and inferior, while others have hemi-optic neuropathy, superior or inferior. We expected that, in patients with hemi-optic neuropathy, PSD had different patterns from bioptic neuropathy due to the lack of overall damage in patients with hemi-optic neuropathy. Our aim was to evaluate the patterns of PSD according to localization of the glaucomatous optic neuropathy. To our knowledge, no previous report has analyzed PSD according to the localization of the glaucomatous optic neuropathy.

## Methods

This retrospective observational study was approved by the Institutional Review Board of Chungnam National University Hospital and was performed in accordance with all relevant requirements of the Declaration of Helsinki. We reviewed the electronic medical records of patients who visited our Glaucoma Clinic at Chungnam National University Hospital between July 2014 and February 2016. Primary open-angle glaucoma (POAG) patients with a best-corrected visual acuity of 20/25 (+0.1 logMAR) or better, who underwent a complete ophthalmic examination including dilated slit-lamp microscopic examination, fundus photography, Cirrus HD optical coherence tomography (Carl Zeiss Meditec, Dublin, CA, USA), and 24–2 Swedish interactive thresholding algorithm (SITA) standard perimetry (Humphrey Field Analyzer II; Carl Zeiss Meditec) were consecutively enrolled. All ophthalmic examinations were conducted within a 6-month period. We excluded patients for whom the reliability of visual field data was poor (>20% fixation loss; >15% false-positive or false-negative data points), as well as those with cortical opacity and/or a subcapsular cataract, posterior capsular opacity, high myopia (< −6.0 D), or any other disease that could secondarily influence visual acuity or the visual field. In addition, patients who had undergone intraocular surgery, other than cataract surgery performed more than 6 months previously, were excluded.

The criteria for a diagnosis of POAG were a glaucomatous optic disc change, a corresponding visual field defect, and an open angle on gonioscopy. Glaucomatous optic disc changes included the characteristic focal or diffuse neuroretinal rim thinning, localized notching, and retinal nerve fiber layer defects that correlated with visual field defects. Glaucomatous visual field defects were defined by two of the following three criteria confirmed on two consecutive visits: the presence of a cluster of three points on a pattern deviation probability plot with a probability < 5%, one of which had a probability < 1%; a PSD with a probability < 5%; or a glaucoma hemifield test result outside the normal limits. All intraocular pressures (IOPs) were medically controlled to below 21 mmHg. One eye of a patient was randomly selected when both eyes satisfied the inclusion criteria.

**Fig 1:**
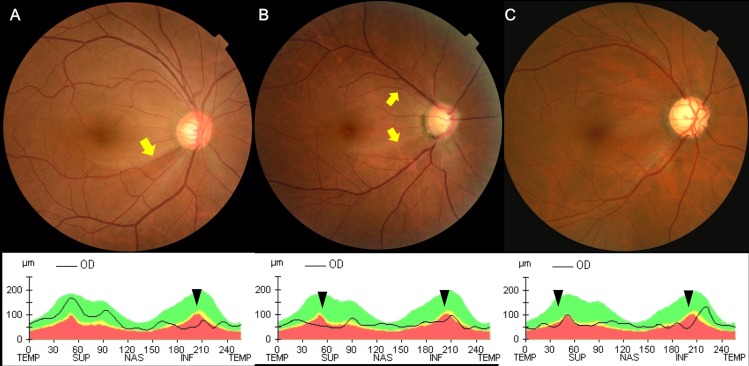
Fundus photographs and optical coherence tomography of representative patients in each group. (A) In a patient with hemi-optic neuropathy, an inferotemporal defect in the retinal nerve fiber layer (RNFL) was evident on fundus photography and inferior thinning of the RNFL was apparent on the peripapillary RNFL thickness profile obtained using Cirrus HD optical coherence tomography OCT. (B) In a patient with bi-optic neuropathy, both superior and inferior RNFL defects were visible on fundus photography, and both RNFL thinnings were confirmed by OCT. The temporal margins of the RNFL defects evident on the fundus photographs of the above two patients, (A) and (B), are arrowed in yellow. (C) In another patient with bi-optic neuropathy, a diffuse RNFL defect was evident on fundus photography and a corresponding region of diffuse RNFL thinning was apparent on OCT. RNFL thinning based on OCT in the above three patients is indicated by black arrowheads.

To classify all patients clearly into a hemi-optic neuropathy group (superior or inferior optic disc damage) and a bi-optic neuropathy group (both superior and inferior optic disc damage), we included only patients in whom the temporal margin of the retinal nerve fiber layer defect on the optic disc was clearly visible on dilated fundus photography, which was confirmed using Cirrus HD optical coherence tomography (OCT). We required that OCT could determine defects from borderline to abnormal (yellow and red color codes, respectively), compared to information in an internal normative database. In some exceptional cases, patients with definite superior and inferior optic disc damage, but with diffuse retinal nerve fiber layer defects without clear temporal margins, were included in the bi-optic neuropathy group. Data from representative patients of each group are shown in Fig 1.

In both patient groups, we analyzed the relationship between MD and PSD, and the differences between MD and PSD (“−MD−PSD”) according to the extent of glaucomatous visual field damage revealed by the MD or the visual field index (VFI, another relatively linear global index provided by the Humphrey visual field analyzer). Because visual field deterioration is expressed as a negative number in MD and as a positive number in PSD, we used “−MD” for convenience when calculating differences between the two parameters (“−MD−PSD”). Thus, if PSD paradoxically improved in accordance with a worsening MD, the “−MD−PSD” value increased. All statistical analyses were performed using SPSS for Windows (ver. 18.0; SPSS Inc., Chicago, IL, USA), and R Language software (http://www.R-project.org) with the segmented R library. We compared the demographics of the two groups using Student’s t-test or Pearson’s chi-squared test. Linear and non-linear regression analyses were used to define the relationship between “−MD−PSD” and MD, and “−MD−PSD” and VFI. For all patients, we speculated that scatter plots of the “−MD−PSD” values would transit from a steep decline to a plateau according to the extent of deterioration of the visual field. Therefore, the scatterplots were additionally fitted to a broken stick regression model with specified tipping points. A *P* value < 0.05 was considered to represent a significant difference.

## Results

A total of 242 patients with POAG met our inclusion criteria; 91 patients had hemi-optic neuropathy and 151 had bi-optic neuropathy. Patient demographics are listed in Table 1. The mean patient ages in the hemi- and bi-optic neuropathy groups were 55.8 ± 12.8 years and 55.8 ± 12.5 years, respectively; these groups included 53 and 100 male patients, respectively. Neither the ages nor the proportions of males differed significantly between the two groups (p = 0.988 and 0.219, respectively). The mean best-corrected visual acuity was 0.0 ± 0.1 logMAR (20/20 Snellen) in both groups. In the bi-optic neuropathy group, the mean MD and PSD were −16.6 ± 6.3 dB and 10.9 ± 3.0 dB, respectively; these values were both significantly worse than those of the hemi-optic neuropathy group, by −6.5 ± 4.6 dB and 6.8 ± 4.5 dB, respectively (both p values < 0.001). Also, the mean VFI of 54.1 ± 21.4% in the bi-optic neuropathy group was significantly worse than that of the hemi-optic neuropathy defect group (84.3 ± 14.1%; p < 0.001). The mean ‘−MD−PSD’ values were 5.7 ± 6.0 dB and −0.3 ± 2.2 dB in the bi- and hemi-optic neuropathy groups, respectively (p < 0.001). None of the reliability indices of the Humphrey visual field test, fixation loss, false-positive, or false-negative rates, differed significantly between the two groups (all p > 0.05). The test duration was significantly longer in the bi- than the hemi-optic neuropathy group (p < 0.001).

**Table 1:** Demographics of patients with hemi-optic or bi-optic neuropathy.

	Hemi-optic neuropathy (n = 91), mean±SD	Bi-optic neuropathy (n = 151), mean±SD	p value
Age (years)	55.8±12.8	55.8±12.5	0.988*
Sex (male/female)	53/38	100/51	0.219^†^
Laterality (right/left)	44/47	79/72	0.596^†^
Best-corrected visual acuity (LogMAR)	0.0±0.1	0.0±0.1	0.199*
Refraction (spherical equivalent, diopters)	-1.8±2.2	-2.5±2.9	0.017*
Intraocular pressure (mmHg)	15.5±2.3	15.6±2.3	0.616*
Central corneal thickness (μm)	534.5±30.3	532.1±32.5	0.728*
Humphrey visual field test			
MD (dB)	-6.5±4.6	-16.6±6.3	<0.001*
PSD (dB)	6.8±4.5	10.9±3.0	<0.001*
-MD-PSD (dB)	-0.3±2.2	5.7±6.0	<0.001*
VFI (%)	84.3±14.1	54.1±21.4	<0.001*
Fixation loss (%)	6.4±6.1	5.8±6.2	0.500*
False positive (%)	2.6±2.7	2.3±3.0	0.421*
False negative (%)	2.6±3.6	3.5±4.6	0.098*
Test duration (s)	299.0±74.9	370.4±74.9	<0.001*
Pupil diameter (mm)	4.7±0.6	4.6±0.7	0.754*

* Student’s *t*-test

^†^ Pearson’s chi-squared test

Hemi-optic neuropathy = patients with superior or inferior glaucomatous optic neuropathy; bi-optic neuropathy = patients with superior and inferior glaucomatous optic neuropathy; MD = mean deviation; PSD = pattern standard deviation; VFI = visual field index; LogMAR = logarithm of the minimum angle of resolution

**Fig 2:**
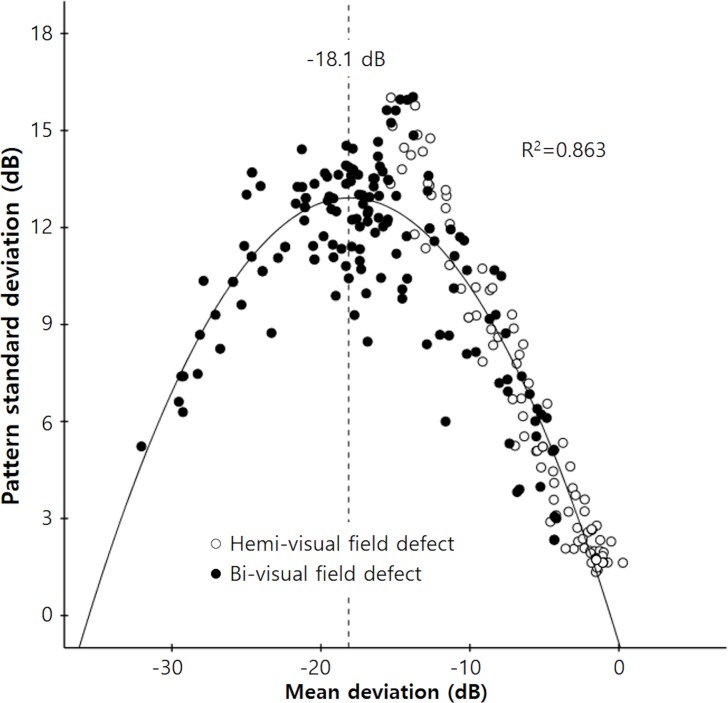
Scatterplot showing the relationship between mean deviation (MD) and pattern standard deviation (PSD). PSD increased as MD decreased from 0 dB to −18.1 dB, and PSD then decreased as MD deteriorated further. Correlations were calculated using the following equation: Y = −0.817 + 1.523X − 0.042X^2^ (r^2^ = 0.863, *P* < 0.001). In the hemi-optic neuropathy group, in which MD values were localized to the right side, the relationship between MD and PSD was more linear in distribution, the correlation coefficient (r) was −0.973, and the linear regression coefficient (β) was −0.965 (*P* < 0.001).

Fig 2 shows the relationships between MD and PSD. PSD increased as MD decreased to −18.1 dB, and PSD then decreased with further deterioration of MD (r^2^ = 0.863, p < 0.001). The hemi-optic neuropathy group, in which damage was localized to the right side, attained a MD of –18.1 dB, and the relationship between MD and PSD showed a more linear distribution, with a correlation coefficient (r) of −0.973 and a linear regression coefficient (β) of −0.965 (p < 0.001).

**Fig 3:**
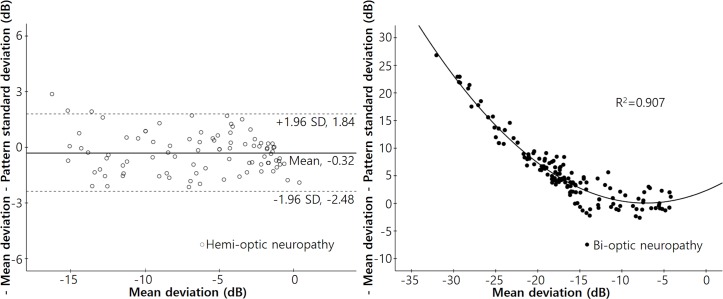
Scatterplots of the differences between negative MD and PSD values with respect to the MDs in each group. Left: A scatterplot of the difference between the negative MD and PSD values (‘−MD−PSD’) with respect to the MDs of patients with hemi-optic neuropathy (either superior or inferior) (r^2^ = 0.019, *P* = 0.191). The mean ‘−MD−PSD’ was −0.32 dB, and the 95% confidence interval ranged from −2.48 dB to 1.84 dB. Right: A scatterplot of “−MD−PSD” in terms of the MDs of patients with bi-optic neuropathy (thus both superior and inferior). The “−MD−PSD” increased significantly as MD decreased. The correlations were calculated using the following equation: Y = 2.320 + 0.632X + 0.044X^2^ (r^2^ = 0.907, *P* < 0.001).

Fig 3 shows the relationships between “−MD−PSD” and MD in each group. In the hemi-optic neuropathy group, the mean value of “−MD−PSD” was −0.32 dB, and the 95% confidence interval ranged from −2.48 dB to 1.84 dB. We found no significant change in “−MD−PSD” by MD (r^2^ = 0.019, p = 0.191). However, in the bi-optic neuropathy group, “−MD−PSD” values increased significantly as the MD decreased, according to the quadratic equation: Y = 2.320 + 0.632X + 0.044X^2^ (r^2^ = 0.907, p < 0.001).

**Fig 4:**
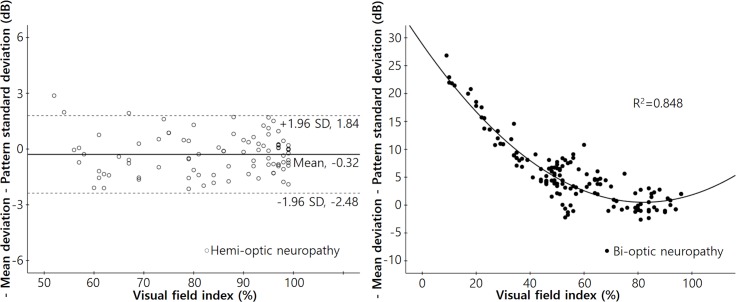
Scatterplots of the differences between the negative MD and PSD values with respect to the visual field index in each group. Left: A scatterplot of the difference between the negative MD and PSD values (−MD−PSD) with respect to the visual field index (VFI) of patients with hemi-optic neuropathy (either superior or inferior) (r^2^ = 0.0001, *P* = 0.916). The mean “−MD−PSD” was −0.32 dB, and the 95% confidence interval ranged from −2.48 dB to 1.84 dB. Right: A scatterplot of the “−MD−PSD” with reference to the VFI in patients with bi-optic neuropathy (thus both superior and inferior). The ‘−MD−PSD” increased significantly as the VFI decreased. Correlations were calculated using the following equation: Y = 28.868 − 0.690X + 0.004X^2^ (r^2^ = 0.848, *P* < 0.001).

Fig 4 shows the relationships between the “−MD−PSD” and the VFI of both groups. In the hemi-optic neuropathy group, we found no significant change in the “−MD−PSD” value as the VFI deteriorated (r^2^ = 0.0001, p = 0.916). On the other hand, in the bi-optic neuropathy group, the “−MD−PSD” value increased significantly as the VFI decreased, according to the quadratic equation: Y = 28.868 − 0.690X + 0.004X^2^ (r^2^ = 0.848, p < 0.001).

**Fig 5:**
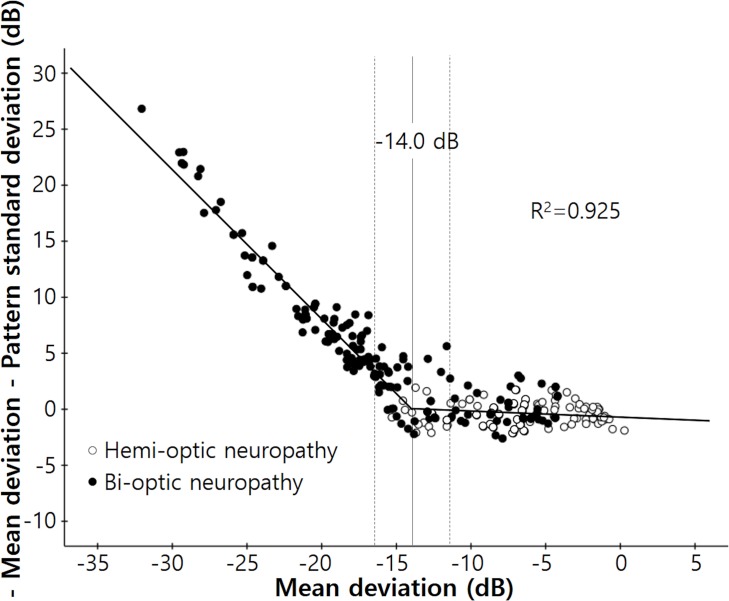
Scatterplot of the differences between negative MD and PSD values with regard to the MDs of the enrolled patients. The “broken stick” model is designated by the black line. The tipping point was −14.0 dB (95% confidence interval, −16.2 ~ −11.8 dB, *P* < 0.001); the slope below the tipping point was given by Y = −18.469 − 1.328X and the slope above the tipping point was given by Y = −0.445 − 0.043X (*P* < 0.0001).

**Fig 6:**
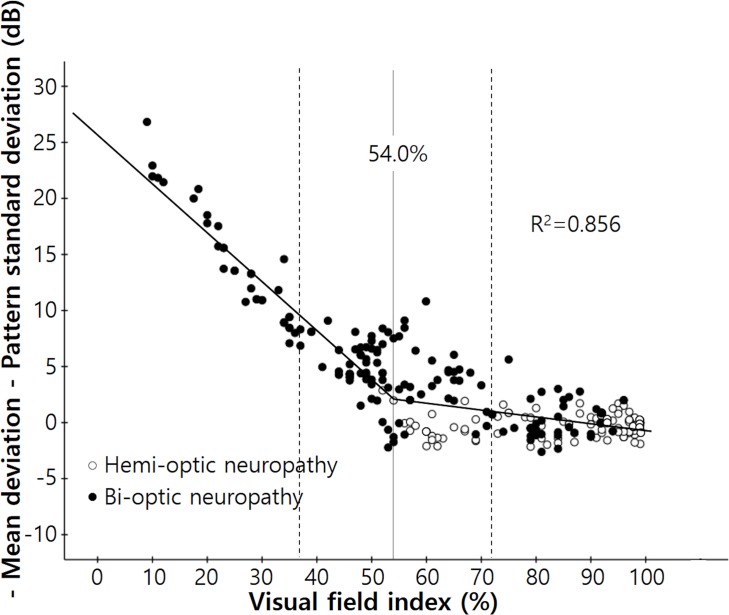
Scatterplot of the difference between the negative MD and PSD values according to the visual field index of enrolled patients. The ‘broken stick’ model is indicated by the black line. The tipping point was 54.0% (95% confidence interval, 36.8~71.2%, *P* < 0.001); the slope below the tipping point was given by Y = 25.747 − 0.437X and the slope above the tipping point by Y = 5.551 − 0.063X (*P* < 0.0001).

The results of the broken stick regression analysis between “−MD−PSD” and MD are shown in Fig 5 for all patients. The MD tipping point was −14.0 dB (95% confidence interval, −16.2 ~ −11.8 dB, p < 0.001) and the slope left of the tipping point was given by Y = −18.469 − 1.328X whereas that right of the point was given by Y = −0.445 − 0.043X. The difference between the slopes right and left of the tipping point was significant (p < 0.0001). A similar relationship was evident between “−MD−PSD” and VFI (Fig 6). The tipping point of VFI was 54% (95% confidence interval, 36.8~71.2%, p < 0.001); the slope left of that point was given by: Y = 25.747 − 0.437X and the slope right of the point was given by: Y = 5.551 − 0.063X. The difference between the slopes left and right of the tipping point was significant (p < 0.0001).

## Discussion

Our data showed that patients with superior or inferior hemi-optic neuropathy had localized visual field defects in the inferior or superior hemi-visual field. PSD showed a strong linear correlation with MD (r = −0.973, β = −0.965, p < 0.001) and “−MD−PSD” was relatively constant (within approximately ± 2 dB) from the stages of early to near-blackout damage of the hemi-visual field (the MD was approximately −15 dB and the VFI was 50%). Blumenthal et al. reported that a visual field of no light perception results with an MD of −31.43 dB [13]. Because glaucomatous depression of visual field sensitivity was confined to a maximum of 26 test points in patients with hemi-optic neuropathy (there were 52 points on the total deviation plot in the 24–2 SITA perimetry), PSD was not expected to be affected by the overall depression that caused PSD reversal. Considering that PSD is less affected by media opacity such as a cataract or a reduction in retinal sensitivity such as high myopia [5, 10, 14–17], in patients with hemi-optic neuropathy, PSD may be a useful index for detecting possible progression of glaucoma that was equal to, or even better than, MD. MD is a useful indicator that shows a linear change with glaucoma progression [3, 4]. Nevertheless, it is directly affected by generalized depression resulting not only from glaucoma but also from media opacity and decreased retinal sensitivity [5–12].

However, in patients with bi-optic neuropathy, “−MD−PSD” gradually increased in line with deterioration of the visual field, and exhibited the ‘U’-shaped graph of a quadratic function. Also, in all patients, the tipping points of MD and VFI (at which points the MD and VFI became significantly associated with an increase in the “−MD−PSD” value) were −14.0 dB and 54%, respectively. These two values are close to 50% damage to the entire visual field. Blumenthal and Sapir-Pichhadze described marked PSD reversal in patients with very advanced visual field defects of MD poorer than −24 dB [13]. However, we found that significant reversal of PSD was already present even in earlier stages of glaucoma. This can be explained by the fact that the diffuse component of a glaucomatous visual field defect can cause an overall decrease in total deviation values, and this in turn can result in a decrease in the irregularity of the hill of vision [18]. Also this was not surprising, because PSD is a form of standard deviation of total deviation values, meaning that regarding the definition of standard deviation, PSD is expected to peak when about 50% of the visual field has gone [19].

Analysis programs of automated static perimetry designed to detect glaucomatous visual field loss are based on certain typical characteristics of glaucoma, including localized damage and differences in the amounts of such damage between regions superior and inferior to the horizontal raphe. However, several previous studies have shown that glaucomatous visual field loss usually accompanies a diffuse component, even in the early stages of disease [18, 20, 21]. In the present study, Fig 2 shows that while the MD and PSD of the hemi-optic neuropathy group exhibited relatively linear distributions, those of the bi-optic neuropathy group were more diffusely distributed even in patients with relatively early-to-moderate damage (the right side of the graph). Our classification criteria meant that almost all patients with diffuse damage sufficient to cause overall depression of the total deviation values were included in the bi-optic neuropathy group (Fig 1).

Ideally, data from all 52 test points of the visual field test should be carefully compared to data from previous tests to detect glaucomatous changes. However, such a comparison requires considerable time and effort. In this respect, global indices such as MD and PSD allow clinicians to determine easily whether visual field deterioration has occurred. Clinicians must always consider the limitations, as well as the unique characteristics, of these global indices. The major advantage of MD is that the change is linear as glaucoma progresses. However, previous studies have reported that MD is overestimated if media opacity such as a cataract is present in addition to glaucoma [6–9, 11, 12]. However, the influence of cataract on PSD is controversial. Some studies have reported that PSD is not affected by cataract [14–17]. Other studies have reported that PSD worsens after cataract extraction, but there is a consensus that PSD is less affected than MD [6, 7, 9, 12].

Our study had certain limitations. First, we cannot completely exclude the possibility that some patients with media opacity were enrolled; the study was retrospective in nature. However, to minimize such errors, we enrolled only subjects with very good visual acuities (better than 20/25; mostly 20/20) and meticulously excluded those with any record of media opacity. Second, the study was cross-sectional in nature. Therefore, a longitudinal study is required to verify our speculation regarding the PSD pattern in accordance with the progression of visual field defects in individual patients with hemi-optic neuropathy.

In summary, in patients with bi-optic neuropathy, a false decrease of PSD usually begins at approximately 50% visual field damage. Therefore, the PSD may be misleading and should not be used in making decisions regarding disease progression. Meanwhile, in patients with hemi-optic neuropathy, PSD shows no paradoxical decrease and shows a linear correlation with MD. We suggest that in glaucoma patients with hemi-optic neuropathy, PSD may be a useful index for detecting the progression of glaucoma that is equal to, or even better than, MD.
